# 

**Published:** 1816-09

**Authors:** 


### CORRESPONDENCE.

Dr. Palmer's Case of diseased Brain; Mr. Bailey's Case, with the Dissection; and Mr. Foy's Case of Melancholia, are unavoidably deferred.

Communications are received from Dr. Von Embden, of Hamburgh.

Dr. Armstrong's Work "Practical Illustrations of Typhus and other Febrile Diseases," has been received. — Lambe's "Addition Reports, &c." are under Review.

